# Re: Hwang et al: Ocular Adverse Events Following COVID-19 Infection: A Self-controlled Case Series Study from the Entire Korean Population

**DOI:** 10.1016/j.xops.2024.100701

**Published:** 2025-01-01

**Authors:** Alan Y. Hsu, Chun-Ju Lin, Huan-Sheng Chen, Yi-Yu Tsai

**Affiliations:** 1 Department of Ophthalmology, China Medical University Hospital, China Medical University, Taichung, Taiwan; 2 School of Medicine, College of Medicine, China Medical University, Taichung, Taiwan; 3 Department of Optometry, Asia University, Taichung, Taiwan; 4 An-Shin Dialysis Center, Excelsior Renal Service Co., Ltd. Taiwan Branch, Taichung, Taiwan

TO THE EDITOR:

We thank Hwang et al^1^ for their insight into uveitis risk after coronavirus disease 2019 (COVID-19) infection. However, we believe their current study design warrants further scrutiny.

The first point we would like to raise is regarding the definition of COVID-19 infection within the study. They identified COVID-19 cases solely based on initial encounter diagnostic codes. Such a study design might introduce potential confounding factors. This approach may explain why they found no significant association between COVID-19 infection and uveitis risk. In studies of this nature, precise COVID-19 diagnosis is critical to accurately assess its connection to outcomes like uveitis. Our study found an increased risk of uveitis following COVID-19 infection from 1 month (hazard ratio [HR]: 1.18, 95% confidence interval [CI] 1.03–1.34) and up to 24 months (HR: 1.16, 95% CI: 1.09–1.22). We used a broader approach that incorporated both diagnostic codes and internationally recognized codes for COVID-19–related serum markers.^2^ A comprehensive approach to defining the exposure of interest—in this case, COVID-19 infection—is crucial, as a less accurate study design can impact findings and reduce their reliability. Studies have shown that COVID-19 diagnoses can often be subject to false positives or even false negatives, further underscoring the importance of accurate identification of infection in retrospective studies.^3^ Future studies by Hwang et al could yield different results if they incorporated these diagnostic elements.

Another key point relates to the lack of propensity score matching and lack of exclusion of certain confounders in the study by Hwang et al. Propensity score matching is a statistical method that allows for the reduction of confounding factors. Hwang et al utilized a study design that lacks propensity score matching, which may have limited the accuracy of their findings and contributed to their lack of observed association between conditions such as COVID-19 infection and complications like retinal vein occlusion and uveitis. In contrast, our retrospective, multicenter study included propensity score matching, creating comparable COVID-19 and non–COVID-19 groups matched by age, gender, and other variables, and found an increased uveitis risk following COVID-19 infection. In another example, a population-based retrospective study by Li et al, which also used propensity score matching to recruit both COVID-19 and non–COVID-19 patients, demonstrated an association between retinal vein occlusion and COVID-19 infection.^4^ Additionally, Hwang et al’s study did not exclude confounding conditions, such as viral hepatitis, human immunodeficiency virus, syphilis, tuberculosis, and autoimmune diseases, which can also influence uveitis risk. Such a lack of exclusion of confounding conditions by Hwang et al again would potentially limit the reliability of their findings.

Finally, we note the relatively short follow-up period in Hwang et al’s study, which limited their analysis of uveitis risk to the 24 weeks following COVID-19 infection. This brief window makes it challenging to make any conclusion about the long-term uveitis risk among patients with COVID-19. In contrast, our study found an increased uveitis risk from 1 month (HR: 1.18, 95% CI: 1.03–1.34) and up to 24 months (HR: 1.16, 95% CI: 1.09–1.22) after COVID-19 infection. We believe a more extended follow-up period might provide a more complete picture regarding any potential association between uveitis and COVID-19 infection.

In summary, we commend Hwang et al for their contribution to understanding the relationship between COVID-19 infection and complications like uveitis or retinal vein occlusion risk. We hope that our observations and comparisons with our study on a similar topic offer useful considerations for future researchers and clinicians interested in the complex association between COVID-19 infection and complications like uveitis and retinal vascular occlusion.
